# Can Asiatic Acid from *Centella asiatica* Be a Potential Remedy in Cancer Therapy?—A Review

**DOI:** 10.3390/cancers16071317

**Published:** 2024-03-28

**Authors:** Michał Wiciński, Anna Fajkiel-Madajczyk, Zuzanna Kurant, Sandra Gajewska, Dominik Kurant, Marcin Kurant, Masaoud Sousak

**Affiliations:** 1 Department of Pharmacology and Therapeutics, Faculty of Medicine, Collegium Medicum in Bydgoszcz, Nicolaus Copernicus University, M. Curie Skłodowskiej 9, 85-094 Bydgoszcz, Poland; michal.wicinski@cm.umk.pl (M.W.); zuzannakurant@gmail.com (Z.K.); dominokurant1@gmail.com (D.K.); 2 Department of Medicinal Chemistry, Faculty of Pharmacy, Collegium Medicum in Bydgoszcz, Nicolaus Copernicus University in Toruń, Dr. A. Jurasza 2, 85-089 Bydgoszcz, Poland; sandra.gajewska@cm.umk.pl; 3 Department of Urology, District Hospital, 10 Lesna Street, 89-600 Chojnice, Poland; marcinkurant@o2.pl; 4 Department of General Surgery, Paluckie Health Center Sp. o.o., Szpitalna 30, 88-400 Żnin, Poland; msousak@wp.pl

**Keywords:** *Centella asiatica*, asiatic acid, triterpenes, cancer

## Abstract

**Simple Summary:**

Cancer is the leading cause of death globally, prompting extensive research into drugs that can enhance survival rates. The aim of our review was to assess the potential of asiatic acid as an anticancer drug, or as a support for existing cancer therapies. In vitro and in vivo studies have shown that asiatic acid activates various molecular pathways and exerts multidirectional effects in an organism. It has been confirmed that metabolic pathways regulated by molecular targets, e.g., TNF-α, NF-κB, PI3K/Akt, VEGF, and others, are affected by asiatic acid and are crucial in tumorigenesis, proliferation, and metastasis. In cancer cells, asiatic acid can decrease gene expression, reduce phosphorylation, and induce apoptosis. The gathered evidence suggests that asiatic acid warrants further investigation as a potential anticancer drug or adjunctive therapy.

**Abstract:**

*Centella asiatica* has been recognized for centuries in Eastern medicine for its pharmacological properties. Due to the increasing prevalence of oncological diseases worldwide, natural substances that could qualify as anticancer therapeutics are becoming increasingly important subjects of research. This review aims to find an innovative use for asiatic acid (AA) in the treatment or support of cancer therapy. It has been demonstrated that AA takes part in inhibiting phosphorylation, inducing cell death, and reducing tumor growth and metastasis by influencing important signaling pathways, such as PI3K, Akt, mTOR, p70S6K, and STAT3, in cancer cells. It is also worth mentioning the high importance of asiatic acid in reducing the expression of markers such as N-cadherin, β-catenin, claudin-1, and vimentin. Some studies have indicated the potential of asiatic acid to induce autophagy in cancer cells through changes in the levels of specific proteins such as LC3 and p62. It can also act as an anti-tumor immunotherapeutic agent, thanks to its inductive effect on Smad7 in combination with naringenin (an Smad3 inhibitor). It seems that asiatic acid may be a potential anticancer drug or form of adjunctive therapy. Further studies should take into account safety and toxicity issues, as well as limitations related to the pharmacokinetics of AA and its low oral bioavailability.

## 1. Introduction

**Figure 1:**
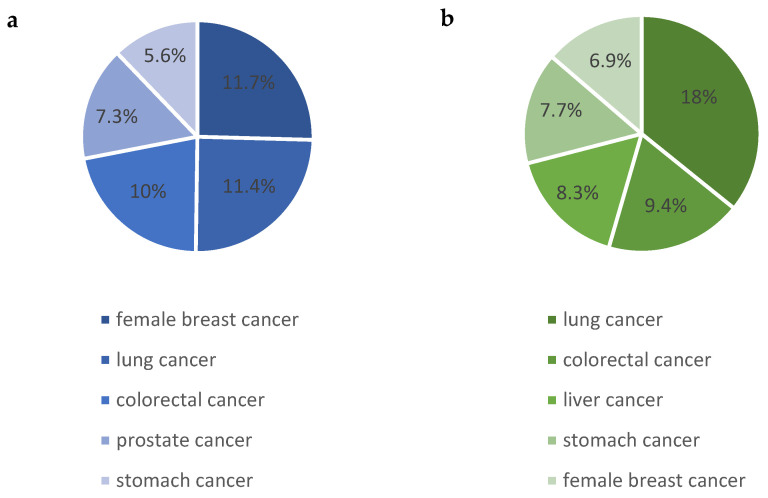
Most commonly diagnosed (**a**) and most lethal (**b**) cancers in the population [1].

Over the years, the increasing significance of cancer as the main cause of death worldwide has become more apparent. In 2019, the World Health Organization (WHO) reported that cancer was the first or second leading cause of death in people under the age of 70 in 112 countries. In addition, it was the third or fourth leading cause of death in a further 23 countries. In 2020, there were around 19.3 million new cancer diagnoses and close to 10.0 million deaths from cancer worldwide [1]. Figure 1 shows statistics on the most frequently diagnosed and most lethal cancers in the population.

Based on current data and trends, it is estimated that over 28 million new cases of cancer will be diagnosed by 2040—an increase of 47% compared to 2020 [1]. These statistics illustrate the importance and necessity of research surrounding new therapies and drugs against cancer. Scientists worldwide are testing known compounds in non-usual areas of application, including cancer. We noticed a trend in research focusing on substances of natural origin as promising compounds for supporting cancer treatment. In our work, we focused on presenting the applications and properties of a plant, *Centella asiatica*, which is becoming more and more popular among scientists engaged in research due to the compounds it contains.

*Centella asiatica* is a plant belonging to the *Apiaceae* family, which has been used for centuries in Eastern medicine. It has been widely known for thousands of years in Asian countries such as India, China, Nepal, Sri Lanka, Madagascar, Malaysia, and Indonesia, and is referred to differently depending on the region. In Chinese medicine, it is known as *gotu kola*; in the Ayurvedic tradition of India, it is commonly known as *mandukparni*; and in Malaysia, it is known as *apegaga*. Other popular names for the plant include Asiatic pennywort, Indian pennywort, and thick-leaved pennywort [2,3,4,5]. Outside Asia, it also occurs in Australia, South Africa, the United States, and Europe, where it is gaining more and more popularity. The popularity and effectiveness of *C. asiatica* are confirmed by historical records of its use, which date back 2000 years in the case of Chinese medicine. In Indian medicine, *C. asiatica* and its extracts were included in the Indian pharmacopeia in the 19th century [6,7,8].

*C. asiatica* has many interesting properties that have encouraged scientists to better understand its effects and potential applications in various fields, due to the compounds it contains. Recently, it has gained commercial interest in the field of dermatology as an ingredient in cosmetics [9]. Ancient records from Chinese traditional medicine speak about its beneficial effects in improving the condition of the skin, and multiple independent studies have additionally confirmed its activity in the treatment of various skin diseases, e.g., lupus, eczema, psoriasis, leprosy, and varicose ulcers [6,10]. *C. asiatica* is also used to accelerate wound healing [11] and in the treatment of neurological diseases [12], such as cognitive impairment and neurotoxicity [13], cardiovascular diseases [7], gastrointestinal diseases [14], diabetes [15], and gynecological diseases [16]. Many studies have also confirmed that this plant has antioxidant properties [17], and anti-inflammatory [12,18] and anti-apoptotic effects [19] in both in vitro and in vivo models.

All the above-mentioned properties of *C. asiatica* result from the large number of chemical compounds that it contains, many of which have pharmacological effects. *C. asiatica* is characterized by a rich content of active substances from various chemical groups such as triterpenes, carotenoids, glycosides, flavonoids, alkaloids, volatile oils, and fatty oils [2,7]. The main group that we would like to pay special attention to here is triterpenes, specifically asiatic acid (AA). These compounds exhibit the previously mentioned properties, but the mechanism of their action is not fully known [20].

The aim of this review is to find an innovative use of *C. asiatica* in the treatment of cancer, or support of cancer therapy. Due to the ever-increasing prevalence of oncological diseases and the insufficient results produced by current treatments, natural substances have become an increasingly important area of research. The natural occurrence of these compounds, and their lower toxicity profiles as compared to currently used chemotherapeutics, contribute to this. Inspired by the above considerations, we wanted to focus on how *C. asiatica,* and specifically its major component, asiatic acid, and its anticancer activities, presents in both in vitro and in vivo studies.

## 2. Triterpenes: Biological Effects and Mechanism of Action

Triterpenes found in *C. asiatica* are considered the main active substances contained in the plant. Our work focuses mainly on asiatic acid, but the group of triterpenes found in the extract also includes asiaticoside, madecassoside, and madecassic acid, which belong to the group of pentacyclic triterpenoids [20].

**Figure 2:**
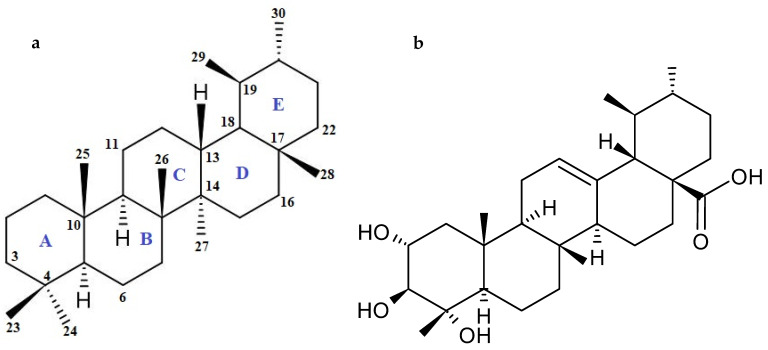
General chemical structure of (**a**) ursane-type formula and (**b**) asiatic acid.

The group of triterpene compounds includes both sterols and triterpenes. In some plants, they accumulate in significant amounts in the form of glycosides (saponins). Asiaticoside and madecassoside belong to the group of saponins (pentacyclic triterpene glycosides), while asiatic acid and madecassic acid are the corresponding aglycones (sapogenins). A hydrophilic sugar chain (glycone) is responsible for the biological activity of saponins. It is a part of the hydrophobic triterpenoid structure aglycone, synthesized via the isoprenoid pathway. Saponins and their aglycones are the most abundant pentacyclic triterpenoids in *C. asiatica.* The basic skeleton of pentacyclic triterpenes contains 30 carbon atoms. Based on the different aglycones, pentacyclic triterpenoids can be divided into four categories, including oleanan, ursane, lupan, and friedelane. Ursane-type triterpenes (Figure 2a) are mostly derivatives of ursolic acid. They have five six-membered rings in their structure, where the A/B, B/C, and C/D rings are all *trans*, and the D/E rings are mostly *cis*. Triterpenoids of this type occur mainly in the free form or as glycosides [21,22,23].

Asiatic acid (Figure 2b) belongs to the ursane category, due to the methyl substitution observed at C19. Due to the presence of hydroxyl groups in its molecular structure, asiatic acid is able to form hydrogen bonds, which influence its activity and enhance the anti-proliferative properties of the compound [21,22,23].

Pentacyclic triterpenoids are characterized by their multidirectional pharmacological effects and high bioactivity. They have immunomodulatory, hepatoprotective, neuroprotective, anti-inflammatory, antioxidant, antibacterial, and antiviral properties. Moreover, they also have the ability to lower sugar levels and blood pressure [5,24,25].

The multidirectional pharmacological properties of AA have been confirmed in in vivo, in vitro, and in silico studies. AA has been shown to regulate the expression of cytokines, chemokines, growth factors, enzymes, signaling molecules, adhesion molecules, apoptosis-related proteins, cell cycle proteins, genes, and various receptors. In addition, asiatic acid has also been demonstrated to modulate the activity of several transcription factors and their signaling pathways [26,27]. Due to this complex pharmacological activity, AA appears to be an ideal drug candidate for many diseases. Table 1 shows examples of AA molecular targets in the organism.

Some of the molecular targets mentioned above are directly or indirectly related to carcinogenesis. Hence, it seems reasonable to examine whether asiatic acid could be a potential drug or adjuvant for cancer therapy.

## 3. In Vitro Studies

According to the WHO, colorectal cancer is the third most common cancer worldwide, constituting approximately 10% of all cancer cases. It is also the second most common cause of cancer-related death worldwide [28]. Hao et al. investigated the effects of AA on the proliferation, migration, and apoptosis of colon cancer cells. For this purpose, scientists used AA dissolved in DMSO (dimethyl sulfoxide) at a concentration of 1 mg/mL, which was further diluted with culture medium prior. A variety of AA concentrations (0–50 µg/mL) were used to treat the cells for 24, 48, and 72 h and significant inhibition of cell growth was observed, with the effect being dependent on time and dose. It turned out that AA changed the morphology of colon cancer cells; they became small and pyknotic, the cytoplasm condensed, and organelles swelled, confirming the cytotoxic effects of AA. Compared to the control group, there was a reduced number of colonies in the study group, indicating reduced proliferation of single cells. In addition, it was proven that AA treatment changed the expression of EMT (epithelial-mesenchymal transition)-interrelated factors in the colon cancer cell groups. Furthermore, it was observed that the expression of E-cadherin was increased, while vimentin and N-cadherin were decreased. Therefore, inhibition of migration of colon cancer cells by AA may be associated with changes in EMT-interrelated factor expression. These factors are well known for their involvement in cancer metastasis [29].

Most interestingly, AA appears to regulate Pdcd4 through the PI3K/Akt/mTOR/p70S6K signaling pathway, which is well-known as a major pathway regulating tumor cell proliferation, apoptosis, and migration. Compared to the control group, the expression of PI3K, Akt, mTOR, p70S6K total protein, and phosphorylated proteins was significantly decreased in AA-treated colon cancer cells. On the other hand, AA increases the expression of Pdcd4 protein in a common cascade [29].

The above findings suggest that AA may cause apoptosis and have an anticancer effect on human colon cancer cells. It seems that AA may be a hopeful agent in colon cancer treatment, which constitutes such a huge challenge in modern medicine.

A study from 2023 conducted by Heise et al. investigated how synthetized 1,5-diazacyclooctane-spacered triterpene rhodamine conjugates affected breast cancer cells. As the experimental triterpenoic acids, they used oleanolic acid, ursolic acid, betulinic acid, platanic acid, and finally asiatic acid, sourced from *C. asiatica* [30]. The findings surrounding asiatic acid interested us the most, because it turned out that the AA–rhodamine conjugate demonstrated the most cytotoxic activity of any of the conjugates in all breast cancer cell lines. The amides themselves in triterpenoic acids showed a low micromolar range of cytotoxicity for all breast cancer lines, but conjugated with rhodamine exhibited an increase in cytotoxicity in all cell lines. It appears that the conjugate acts as a mitocan (compound targeting mitochondria as the final target [31]). At lower doses, it results in inhibition of proliferation, or growth arrest, in breast cancer cells, and at higher doses it induces apoptosis [30].

According to other authors, some triterpenoic acids exhibit very low or no cytotoxicity at all. Rhodamine, which accumulates in mitochondria themselves, is not cytotoxic (up to a concentration of 30 μM), whereas the triterpenoid piperazine-space red rhodamine B derivatives were cytotoxic at a nano-molar concentration. It is likely that the lipophilicity of rhodamine allows it to portion into biological membranes. It also contains a delocalized charge distributed throughout the molecule, which enables passive distribution through a membrane in proportion to an imposed transmembrane electrical potential [32]. 

Therefore, the question arises whether the above effects may be the result of the action of conjugates on mitochondria, where the integration of apoptotic signals takes place. Other authors have suggested that triterpenoic acids linked with rhodamines act as a mitocan through the inhibition of ATP synthesis in mitochondria, which would be consistent with the above considerations [33].

However, we cannot ignore the fact that the cell line HS578T (basal, triple-negative breast cancer cells) turned out to be the most resistant to rhodamine conjugates [30]. That would mean that we have a great base for further research, but it would be worth looking into why some cell lines are less sensitive to the effects of conjugates and where these limitations come from.

Another study suggested that AA from *C. asiatica* may be a promising candidate for drug development in nasopharyngeal cancer. It is necessary to mention that the constitutive activation of STAT3 (JAK/STAT pathway) plays a significant role in nasopharyngeal cancer (NPC) cell proliferation, migration, and invasion. The activation of STAT3 contributes to the cellular invasiveness of NPC, and its overexpression is correlated with advanced stages of NPC. The authors suggested that AA may act on the STAT3 pathway through the inhibition of STAT3 phosphorylation. This inhibition was observed at 40 μM and 20 μM concentrations, and cell viability was reduced as a result; the effect was dose-dependent [18].

The same authors proved that AA induces the expression of caspase-3, and in this way, promotes cell death. In addition, the researchers suggested that asiatic acid may play a role in inhibiting migration due to reduced expression of the mesenchymal markers N-cadherin, β-catenin, claudin-1, and vimentin, which are key in cancer cell migration and metastasis [18].

Undoubtedly, asiatic acid from *C. asiatica* could be a promising option for patients with NPC, and this seems to be supported by data from another study in which scientists examined the cytotoxic effect and mediated mechanism of AA in cisplatin-resistant NPC cells. These cell lines were treated with an increasing concentrations of AA (0, 25, 50, and 75 μM) from *C. asiatica* with or without cisplatin for 24, 48, and 72 h. Cell viability was significantly reduced at concentrations of 50 μM and 70 μM in both cell lines compared to the control group (0 μM). Administration of AA induced apoptosis in examined cells, through both mitochondrial and the death receptor-initiated pathway. It also upregulated expression of two proapoptotic proteins, Bak and Bax. The authors also proved that AA has the ability to increase the expression of caspase-3 (according to the results of previously mentioned research), -8, and -9, and modulates the expression of caspase-9 via phosphorylation of p38 and modulation of the ERK 1/2 pathway in NPC cells in AA induced apoptosis [25].

The consistency of the results from both studies confirms our belief that AA may be a potential therapeutic agent for the treatment of nasopharyngeal carcinomas. Further research is needed to take advantage of this potential.

A noteworthy study from 2024 described the impact of AA on osteosarcoma, a malignant neoplasm that mainly affects young adults. However, not much is known yet about effective therapy options, which has generated even more interest in this research. The authors demonstrated that AA induces apoptosis of human osteosarcoma cells and that this effect increased with rising asiatic acid concentration. Effectively, AA increased the level of Bax expression and decreased BCl2 expression; in this way, AA promoted mitochondrial dysfunction and induced apoptosis. As we mentioned previously, both apoptosis and autophagy are related to tumor cell death, and another positive and surprising discovery was that AA also triggered autophagy in osteosarcoma cells. Using transmission electron microscopy (TEM), scientists examined autophagy by confirming the presence of autophagosomes. After treatment with 40 μM of AA, the TEM images revealed marked autophagotome increases in treated cells. To explain the mechanism of action of AA, scientists subjected the cells to Western blot analysis, which revealed an increased LC3II/I ratio and decreased levels of p62, which are marker proteins associated with autophagy. Moreover, authors examined the levels of PI3K/AKT signaling pathway-related proteins in osteosarcoma cells treated with AA. In these cells, the levels of p-PI3K/PI3K and p-AKT/AKT had markedly decreased. In addition, AA caused an increase in intracellular ROS content. The authors suggested that asiatic acid may inhibit the PI3K/AKT signaling pathway and activate the ROS/MAPK signaling pathway to regulate osteosarcoma cell death mechanisms. In conclusion, this study explored the multi-target and multi-signaling pathways of AA against osteosarcoma [34].

Another promising study regarding the anticancer effects of AA indicated that while this substance does not significantly influence proliferation and cell cycle distribution of renal carcinoma cells (RCC), it does suppress their migration and invasion. The authors suggested that this results from the inhibition of the p-ERK/p-p38MAPK axis by AA and the subsequent down-regulation of MMP-15 (matrix metalloproteinase-15) [35]. Other studies have also described a relation between MMP (matrix metalloproteinase) overexpression and RCC progression and metastasis [36]. It is possible that the connection between MMP expression and AA holds a clue for how to stop the progression of cancer. To confirm this, further research is needed into how AA regulates the expression of MMP.

Interestingly, it has been proven that *C. asiatica* (CA) extracts, in comparison to cisplatin as a positive control, significantly reduced the number of viable cells of oral cancer cell lines, dependent on concentration and the period of incubation. The study revealed that *p*-values were less than 0.05 at concentrations of CA 12.5 µg/mL, 25 µg/mL, 50 µg/mL, and 100 µg/mL, with 24 h, 48 h, and 72 h periods of incubation. The lowest viability of cells was observed after 72 h of incubation with 100 µg/mL concentration of CA extract. However, the authors did not discuss the mechanism of this anticancer effect and it was not determined which of the active compounds of CA is responsible for this effect [37].

Summarizing the above considerations (Table 2), asiatic acid seems to be a brilliant object for research in the field of oncology. In our view, the most attractive properties in this agent relate to its various mechanisms of action, due to which it has anticancer effects. These mechanisms of action include promoting autophagy, stimulating the formation of free radicals, and inducing apoptosis. Additionally, it is important to note how many different cancers the above actions have been described in. Further research should seek to determine the concentrations and chemical forms in which AA shows its greatest anticancer activity.

## 4. In Vivo Studies

In vivo studies provide a lot of important information, not only about the benefits of a drug itself in the treatment of the disease, but also about its safety and potential side effects. Therefore, in this part of our work, we focused on collecting and assessing the effects of *C. asiatica*, especially asiatic acid, on cancers according to in vivo studies.

One such interesting in vivo study was conducted by Kavitha CV. et al. During the study, glioblastoma (GB) cells (U87MG cells) were implanted into mice, both ectopic and orthotropic. After detecting tumor development, a group of 10 mice was divided into two and treated with either 0.9% saline (200 mL) containing 0.01% tween 20 (vehicle control) or asiatic acid with doses containing 30 mg/kg (200 mL in 0.9% saline containing 0.01% tween 20). The second protocol was based on a two-week growth of the xenograft before the implementation of the previously mentioned treatment. Drugs were administered via oral gavage 7 days/week until the end of the 4th week. In the orthotopic xenograft study, GB cells were injected into the brains of 10 animals. In the next step, the group was divided into two equal parts of five individuals. One of these sub-groups was treated as the vehicle control group, and the other was treated with AA (30 mg/kg/twice a day). After a month, the animals underwent magnetic resonance imaging (MRI) of the head, and tumor lesions were measured. The results of the in vivo study showed promise, not only in terms of the effects of AA, but also in terms of safety of use. The xenograft study showed that both groups treated with AA had smaller tumor masses than the control group, even in the group in which the tumor grew for 2 weeks without prior treatment. Very interesting results were also observed in the orthotopic xenograft study. MRI scans showed that tumor growth was observed in 10/10 mice, and in the AA-treated group, tumor volume decreased by 60% compared to the control group. In the AA-treated group, tumor volumes were between 2.22 and 6.22 mm^3^, while in the non-treated group, tumor volumes were between 1.92 and 23.2 mm^3^. Analysis of the test results showed that the effect of anti-GB cells results from the induction of apoptosis in these cells. The probable mechanism of action results from the activation of caspases, modulation of the expression of proteins from the Bcl2 family, and survivin. Moreover, an increase in molecules responsible for the induction of endoplasmic reticulum stress (ER stress) and enhanced intracellular calcium levels was observed in glioma cells, which explains the apoptotic effect of AA [38].

Another study conducted by Li J. et al. examined the effect of AA on tongue cancer cells. Tongue cancer cells (Tca8113) were administered subcutaneously to 8-week-old mice. During the study, when tumor cells grew to approximately 100 mm^3^, some of the tested animals were administered an AA solution in 0.1% DMSO (15 mg/kg/day) or the equivalent of 0.1% DMSO intraperitoneally for 4 weeks. It was observed that both the mass and volume of the tumors were smaller in the group of animals treated with AA. The average tumor weight in the AA-treated group was <0.2 g, while in the control group it was ~0.6 g. Moreover, in the AA group, the average tumor volume was <0.2 mm^3^, while in the control group, it was >0.6 mm^3^. After detailed analysis of samples from both groups using TUNEL staining, it was found that tumors taken from the group of mice treated with AA contained significantly more apoptotic cells than in the untreated group. The mechanism of pro-apoptotic action of asiatic acid from *C. asiatica* in tongue cancer is likely due to the downregulation of Bcl2 family proteins and the upregulation of Bax and cleaved caspase-3 levels. This state of affairs is also the result of the activation of IRE1α/JNK signaling pathway proteins, which cause ER stress and lead to apoptosis of Tca8113 tongue cancer cells. This translates into the percentage of cells that underwent apoptosis in the examined tumors. The apoptotic rate in the DMSO group was <10%, while in the AA group it was over 60%. What caught our attention and the attention of the authors of the study was the small group studied (only 8 animals). This does not change the fact that the effect observed shows promise and leaves room to develop the topic of the impact of AA on tongue cancer, which is closely related to widespread tobacco use all over the world [39].

Tian M. et al. examined the effect of *C. asiatica* leaf extract (mainly AA) on breast cancer in vivo. In the study, they used mice and 4T1 breast cancer cells. The tested mice were administered either AA in 1% DMSO (50 mg/kg) or an equal amount of DMSO alone. After a month of observation and euthanization of the animals in accordance with ethical principles, the authors began their research. During the study, the authors observed not only a decrease in tumor mass and volume in AA-treated animals, as seen in the previously mentioned studies, but more importantly, using immunohistochemistry using CD31 staining, they showed significantly less staining of tumor tissues in the treatment group. Less tissue staining using the mentioned marker might mean less angiogenesis in treated tumors compared to those in the control group. This, of course, may explain the smaller weights (~1 g in AA-treated and ~2 g in untreated) and tumor volumes (~1000 mm^3^ in AA-treated and ~1900 mm^3^ in untreated) obtained from AA-group animals. The promising results from this study are likely due to an AA-induced reduction in the expression of VEGF and the reduction of VEGFR2 phosphorylation, which in the setting of cancer development plays a key role in tumor growth and metastasis. Confirmation of the fact that AA has an effect on the metastasis of breast cancer to the source is provided by scientific research results discovered in mice. It was shown that in mice treated with AA, there was less lung metastasis, and the tumor itself was less invasive in histopathological examinations than in the control group. The fact that the AA-treated group had less meta in the lungs may have occurred as a direct result of the reductions in VEGF and VEGFR2 expression [40].

Another interesting study was conducted by Wu T. et al. The researchers analyzed the effect of AA in different doses. To compare the anticancer effect of *C. asiatica*, and more specifically asiatic acid, one group of mice was administered 5-fluorouracil, a known cytostatic drug from the group of antimetabolites. The test animals were injected subcutaneously with lung cancer cells. After three days, all animals developed tumors. Then the mice were divided into four groups, two of which received AA at a dose of 50 mg/kg or 100 mg/kg, one of which received 5-fluorouracil (5FU), and the final of which was the control group. After 13 days, the animals were euthanized and thoroughly examined. The results of the above activities were shown to be promising, as a reduction in tumor mass was observed in both groups that received AA treatment. In the control group, the tumor weight was ~3 g, while in the group treated with AA at a dose of 100 mg/kg, it was ~1.5 g. It is worth noting that in the mice that were administered 5FU, the antitumor effect was greater than in the case of AA, although it should be mentioned that in this group a decrease in body weight and a lower spleen index were also observed. This means that the cytostatic had a destructive effect not only on the tumor but also on the entire organism, which was not observed in the group treated with AA. The possible proapoptotic mechanisms of asiatic acid might be related to the fact that it provokes the collapse of mitochondrial membrane potential, leading to a rise in reactive oxidative species (ROS) [41].

The next discussed and perhaps most interesting study is one that investigated the potential anti-cancer mechanisms involved in combining asiatic acid with naringenin (NG). Results compiled by Lian et al. demonstrated that the AA + NG combination had a suppressive effect on invasive melanoma (B16F10) and lung carcinoma (LLC) in mouse models. The probable mechanism of action of the combination of these two substances results from the modulation of Smad3/Smad7 signaling in cancer tissues. The above-mentioned effect is justified because hyperactive Smad3 and inhibited Smad7 are observed in developing tumors. Since AA is a Smad7 inducer and NG is a Smad3 inhibitor, this leads to accelerated maturation, increased cell differentiation, and cytotoxicity in NK cells. It is worth noting that in this study the groups of animals were divided into those that were administered AA separately at a dose of 10 mg/kg, NG at a dose of 50 mg/kg, and a combination of the two substances at the same doses as the previous groups. Interestingly, reduced tumor progression was observed in each group compared to the control group, although the best effect was observed in the group administered the AA + NG combination. The results of this study are promising not only because they show the possibility of anti-cancer immunotherapy using asiatic acid, but also prove the safety of its use for organs such as the heart, liver, and kidneys, which is extremely important in the treatment of oncological patients [42].

The above studies have proven that asiatic acid has an inhibitory effect and leads to apoptosis of cancer cells not only in vitro but also in vivo. There are many mechanisms by which the expected effect occurs, including enhancing/quenching the expression of certain genes, influencing an increase in ROS, and inhibiting angiogenesis in tumors [40,43]. Moreover, the positive effects of AA apply to a wide range of cancers such as tongue [39], lung [41], cervical [16], and breast cancers [40,44], as well as glioblastoma [45]. It is also clear that more studies should be performed, especially in vivo studies on larger groups of subjects. Table 3 shows the results of the studies discussed above.

## 5. Challenges and Limitations

Transforming natural products into medicines is currently a major challenge for scientists. Natural compounds are mostly characterized by poor solubility in water, which significantly limits their bioavailability and therapeutic effectiveness and affects their potential clinical use. Likely due to its poor solubility and rapid metabolism, the absolute bioavailability of asiatic acid after oral administration in rats was very low and amounted to 16.25% [46], which suggests the need to work on the pharmacokinetics of potential drugs that apply this ingredient. Therefore, new forms of medicines and dietary supplements that could increase the use of substances of plant origin are constantly being searched for. Interestingly, asiatic acid derivatives have also been investigated for a number of different diseases and have been found to have the potential for therapeutic use. It also appears that chemical alteration of the AA skeleton has a major impact on biological functions; it may not only enhance its anti-cancer activity, but also improve its pharmacokinetic properties [47].

In the case of asiatic acid, it seems that nanodelivery strategies may be a promising approach to increase the therapeutic effectiveness of this compound [44,45]. The preparation of PEGylated nanostructured lipid carriers containing AA appears to be an interesting solution for improving the absorption of asiatic acid in the gastrointestinal tract. Pharmacokinetic studies have shown that this combination prolonged the blood circulation time with a 1.5-fold higher relative bioavailability compared to the standard preparation, which may significantly affect the efficacy of AA in the organism [48]. Studies have shown that the use of nanoparticles with natural substances successfully controlled the growth of cancer cells, which resulted in a better cytotoxic effect. Moreover, the use of the green synthesis method as one of the ways of synthesizing nanoparticles is associated with lower cost, ecology, and easy synthesis in large quantities [49].

It is worth remembering that anti-cancer drugs usually have a very narrow therapeutic index, which involves using appropriate doses to achieve maximum benefit without exposing the patient to the risk of life-threatening toxicity. Unfortunately, adjusting the appropriate dose is not easy, due to the inheritance of specific polymorphisms in genes encoding target proteins and drug-metabolizing enzymes [50]. In the case of cancers, genetic polymorphisms appear to be particularly relevant, so it is essential to consider potential curative therapies from this point of view as well [51]. In studies on asiatic acid, there has been no data included regarding the genetic variability of patients, and clinical data on the larger population are very limited. Developing an effective dose of AA in a specific type of cancer can also be a major challenge.

The safety and toxicity of asiatic acid are also crucial issues that require further research. Most of the conducted studies have utilized in vitro and in vivo models. Precisely planned human trials are necessary to determine the optimal dose and route of administration. Knowing the appropriate dose that has a therapeutic effect in humans would facilitate possible work on the pharmaceutical form of the compound [47].

Studies conducted so far have shown that 250 mg and 500 mg doses of *C. asiatica* extract are safe for humans. Surprisingly, in the human body, the plasma concentration of the major bioactive components achieved better bioavailability compared to the rat model. This is likely due to interspecies differences, specifically the presence of other bacteria in the gastrointestinal tract. One experiment showed that β-glycosidase from human intestinal bacteria can hydrolyze glycosides, whereas animal β-glycosidase does not hydrolyze these glycosides. A limitation of this study was the small study group of participants (n = 11). This shows that further research is necessary on larger groups [52].

Two other studies have indicated that single doses of the extract, in the amounts of 2 and 4 g, are also safe and well-tolerated in humans [53,54]. A review of the literature on this issue shows that there is a lack of research on specific components of the extract, e.g., asiatic acid. In the future, it would be necessary to determine which of the extract components are responsible for the effects caused, and then develop an appropriate dose for single administration, as well evaluating the routes of administration.

Clinical studies using *C. asiatica* extract did not show any serious side effects (for doses of 60–180 mg daily). However, at higher doses, burning pain or skin allergy may occur after injection or local administration. Stomach complaints and nausea have been occasionally reported after oral administration of *C. asiatica* extract [5]. Animal experiments have shown that *C. asiatica* extract has antispermogenic and antifertile effects on the reproductive system of male rats [55]. Additionally, cases of jaundice were reported in three women after taking *C. asiatica* for 20, 30, and 60 days. These women were clinically diagnosed with granulomatous hepatitis, and the symptoms improved after drug discontinuation [56]. Importantly, *C. asiatica* extract and AA inhibit the activity of CYP2C9, CYP2D6, and CYP3A4, which suggests a potential risk of drug interactions [57,58]. There is a lack of clinical data on the toxicity and safety of AA alone in the literature, which is a challenge for future researchers. In addition, a major unknown is the effect of long-term use of asiatic acid and its cumulative properties in the organism, which should also be investigated.

As interest in *C. asiatica* has been ongoing for some time, many studies have already been completed or are in the process of recruiting participants. Of the studies that are currently looking for participants, it is worth mentioning those that will examine the impact of *C. asiatica* on cognitive abilities in Alzheimer’s and Parkinson’s disease, but also those that aim to investigate its strong and beneficial local anti-inflammatory effects. In order to expand knowledge about the abovementioned local effects, the authors of the research focus on, among others, its effect on chronic diabetes foot ulcers, venous foot ulcers, and post-inflammatory hyperpigmentation of skin [59]. However, there are no clinical trials investigating the use of asiatic acid or *C. asiatica* extract in cancer patients.

## 6. Conclusions

In summary, it seems that *C. asiatica* and one of its main components, asiatic acid, could be a very promising agent for the treatment of many conditions. Multiple in vitro and in vivo studies have shown that AA has potential anticancer properties that are worth looking into in future studies.

Studies have shown that asiatic acid activates numerous molecular pathways and has multidirectional effects in the organism. For example, in colon cancer, AA appears to regulate Pdcd4 through the PI3K/Akt/mTOR/p70S6K signaling pathways, which are well known to be the main pathways regulating proliferation, apoptosis, and migration of cancer cells. Furthermore, studies on nasopharyngeal cancer have suggested that AA may interact with the STAT3 pathway by inhibiting STAT3 phosphorylation. In addition, the same study proved that AA induces caspase-3 expression and thus promotes cell death. It also appears that AA may play a role in inhibiting migration due to a reduction in the expression of the mesenchymal markers N-cadherin, β-catenin, claudin-1, and vimentin, which are key in the migration and metastasis of cancer cells. Interestingly, AA can also induce apoptosis in the cells studied, through both the mitochondrial pathway and the death receptor-initiated pathway and can additionally increase the expression of two pro-apoptotic proteins, Bak and Bax. The properties of AA are also significant in decreasing VEGF expression and reducing VEGFR2 phosphorylation, which in the context of cancer development plays a key role in tumor growth, but also in metastasis. Moreover, some studies indicate the potential of asiatic acid to induce autophagy in cancer cells through changes in the levels of specific proteins such as LC3 and p62. The impact of AA on intracellular ROS content also seems to be important in explaining its anticancer effects. Interestingly, it can also act as an anti-tumor immunotherapeutic agent thanks to its inductive effect on Smad7 in combination with naringenin (Smad3 inhibitor), which increases the ability of NK cells to inhibit the progression of invasive melanoma and lung carcinoma in animal models and in vivo.

The pharmacokinetics of the substance, and its low bioavailability after oral administration, may be important limitations to consider in the development of drugs containing AA as part of the formulation. Hence, there is a need to search for new drug formulations to fully exploit the beneficial effects of this substance. There is also a need for clinical trials to provide further important knowledge regarding asiatic acid, particularly its toxicity and safety. Looking into the future, a major challenge will be to consider how to test the effects of AA in cancer therapy without denying effective therapies. This is all the more important as oncology is developing in many directions, and it is not known which of these directions will prove to be the most effective.

In light of these findings, it appears that asiatic acid is suitable for further investigation as a potential anticancer drug or adjunctive therapy.

## Figures and Tables

**Table 1:** Potential molecular targets of asiatic acid (Data from Nagoor et al., 2018 [26] and Ding et al., 2023 [27]).

Molecular Targets	Examples
Cytokines	TNF-α, IFN-γ, IL-1, IL-4, IL-5, IL-6, IL-10
Chemokines	CINC-1/CXCL1, CXCL1/KC, MIP-2, MCP-1
Growth factors	VEGF, TGF, CTGF, FGF, BDNF
Enzymes	AChE, BChE, NOX, eNOS, iNOS, FATP4, ACS, CPT1, ACOX, CYPs, COX-2, LOX, MMP9, MPO, NAG, MAPK
Signaling molecules	ERK, JNK1/2, PGC-1α, PI3K/Akt, AMPK/CREB, mTOR, Akt, Akt/GSK-3β
Adhesion molecules	ICAM-1, VCAM-1
Apoptosis-related proteins	Bcl-2, mdm2, cmyb, Bax, Bak1, Apaf-1, caspases, p53, p38, ATM, DR, Fas, AIF
Cell cycle proteins	cyclin D1
G-proteins	Arf6, Rac1, Cdc42, heat shock proteins 60 and P-gP
Genes	*Col1a1, Tgfb1, Timp1, SREBP-1c, SCD1, HO-1, NLRP3*
Receptors	μ-opioid, PPAR-γ, TLRs
Transcription factors	NF-κB, AP-1, SIRT1, STATs, Nrf2

**Table 2:** Results of in vitro studies.

Authors	Asiatic Acid Form	Observed Effects	References
Heise et al.	1,5–diazacyclooctane-spacered AA-rhodamine conjugate	250 nM of AA conjugate in MDA-MB-231 cells (basal, triple-negative breast cancer cells) significantly inhibits proliferation (under 20% compared to the control cells). 250 nM of AA conjugate in HS578T cells (basal, triple-negative breast cancer cells) → decreases proliferation by about 50%. 500 nM of AA conjugate in HS578T cells reduces cell number by up to 20% compared to control cells.	[30]
Pantia et al.	asiatic acid (97%) prepared as a stock solution of 100 mM by dissolving 4.887 mg of AA in 100 μL of DMSO	Inhibition of STAT3 phosphorylation → reduction of NPC cell viability. Reduction in the expression of mesenchymal markers N-cadherin and β-catenin in claudin-1 → ↓ cell migration and metastasis. Induction of caspase-3 expression → induction of cell death.	[18]
Liu Y. et al.	AA isolated from *C. asiatica* (>98% purity) dissolved in DMSO	Upregulating caspase-3,-8,-9, Bak, Bax expression, phosphorylation of p38, ERK ½ pathway → induction of cell death.	[25]
Hao et al.	AA isolated from *C. asiatica* dissolved in DMSO	Changes in the morphology of colon cancer cells → induction of apoptosis. ↓Expression of E-cadherin; ↑expression of vimentin and N-cadherin → inhibition of migration of colon cancer cells. ↑ Expression of Pdcd4 protein; ↓ Expression of PI3K, Akt, mTOR, p70S6K → induction of apoptosis, anticancer effect.	[29]
He Pang et al.	No data	↓ Expression of BCl2, ↑ expression of Bax → mitochondrial dysfunction → induction of apoptosis. Inhibition of the PI3K/AKT signaling pathway and activation of the ROS/MAPK signaling pathway → ↑ intracellular ROS content. ↑ LC3II/I ratio and ↓ levels of p62 → autophagy promotion.	[34]
Huang et al.	asiatic acid (purity > 98%). The origin stock solution of AA is 100 mM in DMSO solvent.	Inhibition of p-ERK/p-p38MAPK axis and ↓ MMP-15 expression → suppression of migration and invasion of RCC.	[35]

**Table 3:** Results of in vivo studies.

Authors	Asiatic Acid Dose	Observed Effects	References
Kavitha CV. et al.	30 mg/kg/d in ectopic xenograft group or 30 mg/kg/twice a day in orthotopic xenograft group	↑apoptosis ↑activation of caspases ↓ tumor volume ↓ tumor weight ↑ ER stress ↑ intracellular calcium level	[38]
Li J. et al.	15 mg/kg/d	↑apoptosis ↓ tumor volume ↓ tumor weight ↓ downregulation of Bcl2 family proteins ↑upregulation of Bax and cleaved caspase-3 levels ↑ ER stress	[39]
Tian M. et al.	50 mg/kg/d	↑apoptosis ↓ tumor volume ↓ tumor weight ↓VEGF expression ↓VEGFR2 expression ↓ lung metastasis	[40]
Wu T. et al.	50 mg/kg/d or 100 mg/kg/d	↑apoptosis ↓ tumor volume ↓ tumor weight ↑ROS Collapse of mitochondrial membrane potential	[41]
Lian et al.	10 mg/kg/d or 10 mg/kg/d (AA) + 50 mg/kg/d (NG)	↓ tumor volume ↓ tumor weight ↑ NK-cells maturation ↑ NK-cells differentiation ↑ NK-cells anti-tumor cytotoxicity	[42]

## Abbreviations

The following nomenclature is used in this manuscript:

5FU	5-fluorouracil
AA	asiatic acid
AChE	acetylcholinesterase
ACOX	acyl-coenzyme A oxidase 1
ACS	acetyl-CoA synthetase
AIF	apoptosis-inducing factor
Akt	protein kinase B
AMPK/CREB	AMP-activated protein kinase/cAMP response element-binding
AP-1	activator protein 1
Apaf-1	apoptotic protease activating factor 1
Arf6	ADP-ribosylation factor 6
ATM	ataxia telangiectasia mutated protein
Bak1	BCL2 antagonist/killer 1
Bax	Bcl-2-associated X protein
BChE	butyrylcholinesterase
Bcl-2	B-cell lymphoma 2
BDNF	brain-derived neurotrophic factor
CA	*Centella asiatica*
Cdc42	cell division control protein 42 homolog
CINC-1/CXCL1	cytokine-induced neutrophil chemoattractant 1/chemokine (C-X-C motif) ligand 1
*Col1a1*	*collagen*, *type I*, *alpha 1*
COX-2	cyclooxygenase 2
CPT1	carnitine palmitoyltransferase 1
CTGF	connective tissue growth factor
CXCL1/KC	chemokine (C-X-C motif) ligand 1/keratinocyte-derived chemokine
CYPs	cytochromes P450
DMSO	dimethyl sulfoxide
DR	death receptor
EMT	epithelial-mesenchymal transition
eNOS	endothelial nitric oxide synthase
ER	endoplasmic reticulum
ERK	extracellular-regulated kinase
Fas	Fas Cell Surface Death Receptor
FATP4	fatty acid transport protein 4;
FGF	fibroblast growth factor
GB	glioblastoma
GSK-3β	glycogen synthase kinase 3β
*HO-1*	*heme oxygenase-1*
ICAM-1	intercellular adhesion molecule-1
IFN-γ	interferon gamma
IL-1	interleukin 1
IL-4	interleukin 4
IL-5	interleukin 5
IL-6	interleukin 6
IL-10	interleukin 10
iNOS	inducible nitric oxide synthase
JNK1/2	c-Jun N-terminal kinase ½
LC3	Microtuble-associated protein light chain 3
LLC	lung carcinoma
LOX	lipoxygenase
MAPK	mitogen-activated protein kinase
MCP-1	monocyte chemoattractant protein-1
mdm2	mouse double minute 2 homolog
MIP-2	macrophage inflammatory protein 2
MMP9	matrix metallopeptidase 9
MMP15	matrix metallopeptidase 15
MPO	myeloperoxidase
MRI	magnetic resonance imaging
mTOR	mammalian target of rapamycin
NAG	N-acetylglutamate
NF-κB	nuclear factor kappa B
NG	naringenin
*NLRP3*	*NLR Family Pyrin Domain Containing 3*
NOX	NADPH oxidase
NPC	nasopharyngeal cancer
Nrf2	nuclear factor erythroid 2-related factor 2
Pdcd4	Programmed Cell Death 4
PGC-1α	peroxisome proliferator-activated receptor γ coactivator 1α
P-gP	glycoprotein P
PI3K/Akt	phosphoinositide 3-kinase/protein kinase B
PPAR-γ	peroxisome proliferator-activated receptor gamma
p-p38MAPK	Phosphorylated-p38 mitogen-activated protein kinase
Rac1	Rac family small GTPase 1
RCC	Renal Carcinoma Cells
ROS	reactive oxidative species
*SCD1*	*stearoyl-CoA desaturase-1*
SIRT1	sirtuin 1
Smad3	Mothers against decapentaplegic homolog 3
Smad7	Mothers against decapentaplegic homolog 7
*SREBP-1c*	*sterol regulatory element binding protein-1*
STATs	signal transducer and activator of transcription proteins
TGF	transforming growth factor
*Tgfb1*	*transforming growth factor beta 1*
*Timp1*	*tissue inhibitor of metalloproteinases 1*
TLRs	toll-like receptors
TNF-α	tumor necrosis factor alpha
VCAM-1	vascular cell adhesion molecule 1
VEGF	vascular endothelial growth factor
WHO	World Health Organization
